# Why so many Agta boys? Explaining ‘extreme’ sex ratios in Philippine foragers

**DOI:** 10.1017/ehs.2019.4

**Published:** 2019-05-30

**Authors:** Abigail E. Page, Sarah Myers, Mark Dyble, Andrea Bamberg Migliano

**Affiliations:** 1 Department of Anthropology, University College London, London, UK; 2 Department of Population Health, London School of Hygiene and Tropical Medicine, London, UK; 3 Jesus College, University of Cambridge, Cambridge, UK; 4 Department of Anthropology, University of Zurich, Zurich, Switzerland

**Keywords:** Biased sex ratios, female-biased infanticide/neglect, male-biased mortality, hunter–gatherers, Agta

## Abstract

Male-biased sex ratios have been observed in multiple small-scale societies. Although intentional and systematic female-biased mortality has been posited as an explanation, there is often a lack of ethnographic evidence of systematic female neglect and/or infanticide. The Agta, a foraging population from the Philippines, have a skewed sex ratio of 1.29 (129 males per 100 females) aged 15 years or under. We hypothesised that this skew was *not* caused by greater female deaths, but due to an adaptive response, where more males were produced at birth in reaction to high male-biased extrinsic mortality. To test this hypothesis we utilise census, childcare and mortality data from 915 Agta. The Agta's sex ratio is significantly male-biased in the <1 (*n* = 48, 2:1) and 1–5 year (*n* = 170, 1.39:1) age cohorts; however, we find no evidence of systematic female neglect in patterns of childcare. Furthermore, the sex ratio decreases over cohorts, becoming balanced by the end of the juvenile period, owing to significantly higher male mortality. Taken together, these results are not supportive of female infanticide or neglect, and instead suggest an adaptive mechanism, acting *in utero* as a response to male-biased juvenile mortality, following Fisherian principles of equalising parental investment.

## Graphical abstract

**Figure:**
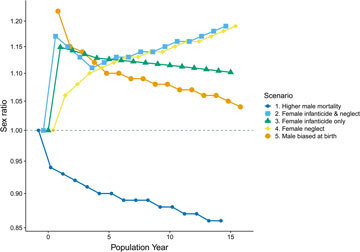

## Introduction

Many species of sexually reproducing organisms have biased sex ratios, including mammals, birds, frogs and lizards (Navara 2013; West *et al.* 2002), varying both at the species and at the population level. Humans are no exception; worldwide, more boys than girls are born, with the sex ratio at birth in 2016 standing at 1.07 (United Nations 2017). Yet cross-cultural variation exists, with African countries tending towards lower sex ratios (Mace and Jordan 2005) – of the 50 counties for which data is compiled, the sex ratio at birth in 2017 ranged from 1.01 to 1.06, with the majority (*n* = 26) at 1.02 (CIA 2018). Much of the literature on highly skewed sex ratios in human populations cites infanticide or neglect of one sex, usually females, as the causal explanation (Hesketh and Xing 2006). Although such explanations are undoubtedly valid in certain contexts, the fact that sex ratios vary in other species without necessitating sex-biased infanticide and/or neglect suggests that the mechanisms in human populations need not be *exclusively* behavioural. Furthermore, the non-human literature also indicates an evolutionary or functional rationale for sex ratio manipulation which should also apply to humans; previous tests, largely in WEIRD (Western, educated, industrialised, rich and democratic) populations replete with potential confounds (Beckerman et al. 2017), however, have found mixed support for this (Cronk 2007; Lazarus 2002). Here we set out to explore the underlying explanation – in terms of both evolutionary function and potential mechanism – for the male-biased sex ratio in the Palanan Agta, a foraging population from the Philippines.

Fisher (1930) proposed that because, in sexually reproducing species, every individual has one parent of either sex, within a population parents should invest equally in sons and daughters. Thus, when the costs of raising sons and daughters are equal, in a population with a biased sex ratio the parents of the rarer sex will have a selective advantage, driving the sex ratio back to a balanced state, producing an evolutionarily stable strategy of a 1:1 sex ratio. However, when the relative costs of the sexes are unequal, parental investment across the sexes is equalised when females produce more of the cheaper sex (McElreath and Boyd 2007). Where the mortality of one sex is consistently higher than the other before the end of parental investment, the higher-mortality sex will not receive their full share of investment (Fisher 1930). For every 100 of the higher-mortality sex successfully reared, the total investment is the sum of the investment in those surviving to independence *and* that in those who died prematurely. Consequently, the average cost of each of this sex reared to independence is greater, but less for each one born, than it is for members of the opposite sex at the corresponding stages. By adjusting the sex ratio *at birth* in favour of the higher-mortality sex, parents can equalise their investment (Clutton-Brock et al. 1982; Fisher 1930; Skogland 1986). Additionally, where the competition or cooperation between kin differs by sex, the cost–benefit ratio of producing one sex over another is also expected to vary. Parents are thus expected to increase the number of the most ‘helpful’ sex, and thus, optimise their inclusive fitness (Hamilton 1967; Silk 1983). Finally, offspring sex ratios may also be adjusted depending on the condition of the mother (Trivers and Willard 1973). Mechanisms enabling sex ratio variation are predicted to be selected for when the benefits to parental fitness of this behaviour outweigh the costs (West et al. 2002), and the activation of such mechanisms is expected to be context dependent (Sieff et al. 1990).

Early ethnographers documenting hunter–gatherers, particularly those working in the Arctic, published accounts of extensive sex-selective infanticide resulting in high (male-biased) sex ratios (Rasmussen 1931; Weyer 1932). For instance, the juvenile (under 16 years) sex ratios of 10 Inuit populations from 1880 to 1930 ranged from 1.05 to 2.24 (Smith and Smith 1994). Subsequently, male-skewed juvenile sex ratios have been widely documented in foragers (Table 1).

**Table 1.** The juvenile sex ratios and the sample sizes on which they are based as reported by Hewlett (1991)^a^ and Headland (1989)^b^ – the Palanan Agta data are our own.^c^ Apparently male-biased sex ratios are in bold

Population	Juvenile sex ratio		Population	Juvenile sex ratio	
	#m/#f	Ratio		#m/#f	Ratio
Ache^a^	55/36	**1**.**53**	Agta (Casiguran 1977)^b^	124/102	**1**.**22**
Agta (Casiguran 1984)^b^	125/86	**1**.**45**	Agta (Palanan)^c^	281/217	**1**.**29**
Aka^a^	160/139	**1**.**15**	Batak^a^	77/72	**1**.**07**
Batek^a^	31/18	**1**.**72**	Bihor^a^	30/18	**1**.**67**
Chenchu^a^	182/195	0.93	Central !Kung^a^	49/43	**1**.**14**
Efe^a^	67/63	**1**.**06**	Groot Eylandt^a^	46/36	**1**.**28**
Hill Pandaram^a^	142/133	**1**.**07**	Inuit^a^	64/57	**1**.**12**
Mbuti^a^	101/84	**1**.**19**	Northern !Kung^a^	64/79	0.81
Ongee^a^	9/15	0.60	Paliyan^a^	9/13	0.69
Siriono^a^	28/32	0.87	Xavante^a^	172/139	**1**.**24**

These sex ratios are often seen as ‘extreme’ and, as such, taken as evidence of infanticide and/or systematic female neglect (Hewlett 1991; Hewlett et al. 2000; Hurtado and Hill 1987; Sharp 1940; Smith and Smith 1994), implying that it is possible to establish infanticide from sex ratios alone (Kelly 2013). However, while female-biased infanticide and neglect have been documented in larger-scale societies (United Nations 2017; Reza et al. 2001; Watts and Zimmerman 2002), and some non-egalitarian foragers [Inuit populations from the northern Arctic (Balikci 1970; Irwin 1989; Rasmussen 1931; Weyer 1932); the Aché of eastern Paraguay (Hill and Hurtado 1996; Hill and Kaplan 1988); some Australian Aboriginal groups (Rose 1960; Sharp 1940); and the Cuiva of Venezuela (Hurtado and Hill 1987)], they remain ethnographically rare in egalitarian foragers. Amongst the Agta hunter–gatherers from the Philippines, sex-biased infanticide has never been witnessed, by our group or others (Headland 1989; Minter 2018 personal communication). Such an explanation is also at odds with the egalitarian social and political structure of many immediate-return foraging populations (Dyble et al. 2015; Kelly 2013). Indeed, ethnographers, in a range of foraging populations worldwide, frequently find no sex bias in investment by parents or a stated preference between the sexes (Eder 1987; Gardner 1988; Hewlett 1993; Morris 1982; Turnball 1961; Yengoyan 1970).

In attempting to explain male-biased sex ratios in foragers, evolutionary anthropologists have typically explored the functional question of ‘why are males preferred to females?’, arguing that more sons increase the relative fitness of their kin as males have higher mean calorific productivity (Hewlett 1991; Hewlett et al. 2000; Hill and Hurtado 1996; Smith and Smith 1994). While calorific contributions are an indicator of cooperation, we cannot quantify a measure of cooperativeness by only exploring male-dominated food production. For instance, cooperation in breeding is extensive in foragers (Crittenden and Marlowe 2008; Kramer 2010; Meehan 2005) and unmarried female siblings frequently help raise their siblings more than unmarried males, as is in the case in the Palanan Agta (Page 2016). Thus, we might equally expect parents to prefer females (Emlen et al. 1986; Mace and Jordan 2005). Furthermore, cooperation is not confined to caloric contribution and allocare but extends across a number of domains which are substantially harder to quantify. It is incredibly complex to measure and reliably compare contributions to parental inclusive fitness of the sexes, as in part it will depend on one's operationalisation of ‘help’.

The relative aid provided by the sexes is not necessarily the only reason sons and daughters differ; as noted, sex-biased extrinsic mortality (i.e. mortality beyond parental control) alters the relative costs of the sexes (Fisher 1930). In many avian and mammalian species, males are frequently the less viable sex (Clutton-Brock and Iason 1986; Promislow 1992) and male-biased sex ratios at birth have been argued to be the result of an optimal strategy when males are vulnerable to mortality before the end of parental investment (Austad and Fischer 2016; Clutton-Brock et al. 1982; Skogland 1986). A trend for male-biased mortality extends to humans, even in low-mortality contexts (Drevenstedt et al. 2008; Wells 2000). This has been suggested to underlie the common human at-birth sex ratio of 1.05–1.07 (Mace and Jordan 2005), indicating the existence of biological sex ratio manipulation mechanisms in humans. Arguably then, the high juvenile sex ratio in many hunter–gatherers may be the product of a facultative alteration, amplified by higher mortality risks (Gurven and Kaplan 2007; Hill et al. 2007). One need not default to a behavioural explanation of infanticide/neglect when sex ratios seem ‘extreme’.

The hunter–gatherer literature has largely overlooked biological mechanisms, proceeding on the assumption that the sex ratio at birth is fixed and parents must augment their fitness after sex has been determined (Maynard Smith 1980). However, post-birth infanticide and selective neglect are energetically inefficient compared with a biological mechanism capable of altering the primary (at conception) or secondary (at-birth) sex ratio. Therefore, it is reasonable to suggest there would have been a selective advantage in the human lineage of both being able to regulate the optimal sex to bear in a given environment and in terminating investment prior to birth, as suggested more broadly by West et al. (2002). This is certainly the case in other taxa; birds, for instance, have been found to show sex-selective re-absorption of eggs after conception (Komdeur et al. 1997). In mammals, biased prenatal mortality owing to maternal nutritional (Labov et al. 1986; McClure 1981) and social stress (Silk 1983) and increased hormone levels at the time of conception (James 1986) have all been associated with at-birth sex ratio skews. In humans, the use of medical records from the 2011 Japanese earthquake and tsunami – which dramatically increased extrinsic mortality risks – indicate the existence of a biological mechanism(s) which both reduced implantation rates of male zygotes and triggered miscarriages of male foetuses later in gestation (Catalano et al. 2013).

Foraging populations are often small, and what may appear to be an ‘extreme’ sex ratio may be an artefact of the limited number of observations. Therefore, it is first essential to demonstrate that sex ratios are in fact statistically male-biased. Here, we do so utilising census and reproductive histories. We detail at-birth, infant and juvenile sex ratios in the Palanan Agta. We hypothesise that female neglect and/or infanticide are *not* the mechanisms underlying the Agta's high sex ratio. Instead, we argue that these trends may originate from an adaptive adjustment of the sex ratio at birth in response to male-biased extrinsic mortality during childhood and adolescence. From this hypothesis, we test three predictions. In *Prediction 1*, firstly, we explore the sex ratios across age cohorts using proportion tests to assess the prediction that sex ratios decrease over childhood, rather than becoming increasingly male-biased as expected when mortality is female-biased (illustrated by plotting five differing mortality regimes). In *Prediction 2*, secondly, we look at parental care data relating to 35 children to test the prediction that there is no evidence of systematic female neglect. In *Prediction 3*, finally, we conduct proportion tests and hazard analysis on childhood mortality data to test the prediction that mortality prior to the end of parental investment is male, not female, biased.

## Methods

Data collection occurred over two field seasons from April to June 2013 and February to October 2014. We visited 20 Agta camps two to three times to collect both demographic and childcare data; during this time we met 915 Agta. No formal sampling techniques were used; as this was a small population, we were able to census and interview the majority of the population. This research and fieldwork was approved by UCL Ethics Committee (UCL Ethics code 3086/003) and carried out with permission from local government and tribal leaders in Palanan. Informed consent was obtained from all participants, and parents signed the informed consents for their children (after group and individual consultation and explanation of the research objectives in the indigenous language).

### The Agta

The Agta reside in the Northern Sierra Madre Natural Park in the northeast of Luzon, a protected area that consists of a mountainous tropical rainforest and includes the coastal beaches, coral reefs and marine eco-system of the Pacific Ocean. Mobility both within Palanan and between Palanan and neighbouring areas makes it difficult to calculate the exact population of Agta in Palanan at any one time, although Minter (2010) estimated a population of 1000. The Agta rely heavily on foraging modes of subsistence (76.5% of out-of-camp economic activity) vs non-foraging activities (23.5%), but this varies by camp and location (Page et al. 2016). Riverine and marine spearfishing provides the primary source of animal protein, supplemented by inter-tidal foraging and hunting and the gathering of wild foods, as well as low-intensity cultivation (Dyble et al. 2016; Minter 2010; Page et al. 2016). The Agta have also long resided with neighbouring farming populations, trading meat for rice and, historically, tubers (Peterson 1978). The Agta practise serial monogamy and have a total fertility rate of 7.7 (Page et al. 2016). Infant and childhood mortality rates are also high, with 38.9% of offspring reported to have been born dying before the age of 16 years (Page et al. 2016); 19% of children died before reaching 1 year old (reaching 36.9% if stillbirths and miscarriages are included), while 13.9% died between ages 1 and 5 years, and 6% died aged 5–15 years.

### Reproductive histories and juvenile mortality data

We (A.E.P. and M.D.) interviewed and collected reproductive histories from all adult women encountered (*n* = 161), producing a record of all reported living (*n* = 632) and deceased (*n* = 210) children. Underreporting of infant mortality is always a possibility as mothers may be unwilling to report a child who later died (Ellison et al. 2000). To limit the issue of underreporting, we asked mothers to report all children born, including miscarriages or stillbirths, in the order they were born and their birth interval. If there was a large interbirth interval between any two children we would enquire if there was a specific reason, which often prompted a mother to report a deceased child. Furthermore, genealogical interviews were conducted with all adult members of the communities, therefore missing children would become evident when, for instance, a wife's and husband's reports did not match, or did not match that of any other reliable close kin member (generally a co-residing sister or mother). In this case we would go back and check with mother to resolve the issue. There is some evidence of older women underreporting births and, by extension, deaths, as evident in differences in the completed fertility rate of mothers who had recently finished their reproductive lifespan (aged 40–55, *n* = 31, 8.5 reported children ever born) compared with older mothers (*n* = 12, 6.5 reported children ever born). Nonetheless, our mortality figures fall into line with previous research findings among immediate-return foragers (Kelly 2013).

If a child was reported to have died, we enquired about the cause of death and their approximate age at death. Cause of death was documented for 155 deceased children aged 15 years or under at time of death. Deaths were classified as either disease-related (*n* = 123) – including malaria, hepatitis, respiratory trait infections, gastro-intestinal disease, measles and malnutrition – or non-disease causes (*n* = 32) – including accidents, violence and environmental events such as typhoons. Ages were established for the entire population using relative age lists and a Bayesian methodology which accounts of the inherent uncertainty in aging a hunter–gatherer population with no concept of calendar years (see Diekmann et al. 2017 for full details). Estimated survival times were calculated for 623 children for whom sex and date of birth and, where applicable, date of death were available and whose mothers were interviewed on multiple camp visits (increasing data validity).

While in the field we also documented a number of ‘witnessed’ births (*n* = 16). These births do not reflect *all* births which occurred during 2013–2014, as some women gave birth before we arrived and others were yet to give birth when fieldwork ended and we did not know of all pregnancies, thus we cannot put too much weight on analysing this cohort. Nonetheless, it is important to include it as it is our only measure of a ‘true’ at-birth sex ratio, as opposed to an at-birth sex ratio calculated from reported births, and also speaks directly to the question of sex-biased infanticide.

### Parental investment observations

Focal follows were conducted on 35 children [17 infants aged 0–1.9 years (average 0.87 ± 0.55) and 18 toddlers aged 2–5.9 years (average 3.9 ± 1.2)] for 9 h per child to assess parental investment. These intensive observations were conducted using focal sampling techniques detailed by Meehan (2005) and further details and descriptive statistics can be found in the Supporting Information (see Table S1). The following parental activities were documented and classified into either ‘high’ or ‘low’ investment: high – carrying, feeding, grooming, medical attention, teaching, playing; low – talking to, watching, being in proximity. For the 35 children (13 = female) observed this produced 124,701 dyadic child–carer data points, of which 24,949 were between mothers (*n* = 23) or fathers (*n* = 22).

### Analysis

#### Describing sex ratios in the Agta

We plot census sex ratios across age cohorts and assess whether: (1) the apparent male bias across juvenile age cohorts (in the <1, 1–5, 6–15 and 16–25 year olds) is significantly different from 1:1 using two-tailed binomial exact tests; and (2) the male bias in the ‘witnessed’ births and the <1 cohort is greater than what demographers consider to be the ‘normal’ sex ratio of 1.05–1.07 (Hesketh and Xing 2006) using one-tailed binomial exact tests. We report the 95% confidence intervals (CI) or lower-bound CI (LCI) for one-tailed tests; for full results see Table S2. We also present the sex ratios of individual mothers who had two or more children, in acknowledgement that population-level analyses may mask individual variation (West et al. 2002), and the sex ratio of yearly births cohorts.

#### Prediction 1 – sex ratios become decreasingly male biased

*Proportion tests using census data.* To test *Prediction 1*, we assessed the changes in the sex ratio over the period of parental investment, considered to end during the 16–25 year old cohort based on the average age at marriage being 19 for females and 22 for males (Minter 2010; Smith and Smith 1994). We performed Pearson chi-squared (*χ*^2^) tests to assess whether the proportion of males differed significantly between cohorts, assessing differences in distribution for all combinations of the following cohort groupings: <1, 1–5, 6–15 and 16–25 year old cohorts.

##### Illustrating differential mortality patterns on sex ratios trends

Different patterns of mortality between the sexes should leave a unique signature, traceable in the relative distribution of the sexes across age cohorts of a population. By creating hypothetical data for five different demographic scenarios we illustrate how sex ratios respond to different mechanisms known to influence sex ratios. Comparing these patterns with that seen in our census data, we seek to gauge the plausible mechanism underlying the sex ratios observed in the Agta. Five sex ratio distributions were created by subjecting 1000 hypothetical, simultaneous births to five demographic scenarios over 15 population years [mirroring the cut-off commonly used for differentiating juvenile and adult mortality in small-scale societies (Hewlett 1991; Kelly 2013)].

This exercise is intended to be purely illustrative, highlighting the alternative trajectories of sex ratios as a result of differential juvenile mortality by sex and, as such, the specific sex ratios employed are irrelevant; however, the statistics (Table 2, SI methods) chosen for each mortality regime are within the bounds of those documented in ‘real-world’ forager populations (Blurton Jones 2016; Kelly 2013). For the sake of this illustration we make the simplifying assumption that, where purely behavioural mechanisms underlie sex ratio patterns, the sex ratio at birth will be balanced.

**Table 2.** Mortality statistics applied to a hypothetical population of 1000 births at year 0 (see Figure 2). The scenarios are intended for illustrative purposes only; however, the statistics are within the bounds of those documented for extant foraging populations. For instance, the average percentage of juveniles dying across 27 societies by age 16 is 35.3% (Kelly 2013: 201); in our sample 38.9% died. Sex differences in mortality have been found to range up to 14% (Kelly 2013). Mortality rates are also set to be highest in early life, as is typical of non-industrialised populations (Blurton Jones 2016). For example, in the Hadza 51.8% of deaths occurring by year 15 had occurred by year 1 and 80.5% by year 4; the equivalent figures for the Agta are 48.8% and 84.5% (Page et al. 2016). At birth sex ratios have been previously documented as being as high as 122 (Hewlett 1991).

Scenario	Overall juvenile mortality by year 15 (%)	Sex ratio at birth	Differential mortality by sex			Rationale behind the statistics
			Percentage of overall mortality by year 1 (m/f)	Percentage of overall mortality by year 4 (M/F)	Percentage of overall mortality by year 15 (m/f)	
*Mechanism – none*						
1. Higher male mortality	36	100	50/50	75/75	41/31	‘Naturally’ higher male mortality, no compensatory mechanisms = *baseline* condition
*Mechanism – behavioural*						
2. Female infanticide and neglect	41	100	50/65	75/75	36/46	Compared with *baseline* – males preferentially treated, so more survive; a lot more females die owing to neglect, with majority of excess deaths in the first year
3. Female infanticide only	43	100	50/65	75/90	41/46	Compared with *baseline* – females experience excess death in first year, male mortality unaffected
4. Female neglect	41	100	50/50	75/75	36/46	Compared with *baseline* – males preferentially treated, so more survive; a lot more females die owing to neglect
*Mechanism – biological*						
5. Male biased at birth	36	120	41/31	50/50	75/75	Compared with *baseline* – more males born, otherwise the same

#### Prediction 2 – females do not receive less parental investment

##### Multilevel analysis of observational childcare data

To test the prediction that females do not receive differential levels of care we used the focal follow data to create three investment measures: (a) *high-investment interactions*; (b) *low-investment interactions*; (c) *total interactions* (sum of high and low) for each child–parent dyad. Mothers and fathers were assessed both in combination and separately, giving three sources of investment: (a) *parental investment*; (b) *maternal investment*; and (c) *paternal investment*. There was slight variability in the total duration of observations; to standardise this, the data were transformed into a proportion of the total number of observations, theoretically ranging between 0 (no interaction) and 1 (constant interaction). For a breakdown of the average proportion of investment by type and source see Table S1. The proportion of interactions acted as the outcome variable and child sex was the predictor variable (reference = male). Multilevel models (using the R package *lme4*) were used to capture the non-independence of data points as children were clustered into households. When ‘paternal high-investment interactions’ were the outcome variable, the household variance was reduced to 0, indicating that the multilevel structure was not parsimonious; in this instance, parental investment was assessed using a single-level multivariable regression. Given the small sample size, the models were kept as simple as possible owing to power considerations. Therefore, each model contained a term for child sex and the child's age.

##### Wilcoxon rank sum tests within families

While the sample is small, we explored whether brothers and sisters received differential investment (sibling sets *n* = 5, children *n* = 12) using Wilcoxon rank sum tests. This additional analysis was to examine whether parents invested more in sons compared with daughters when they had a daughter, as arguably parents with *only* sons or *only* daughters may not show differential investment patterns.

#### Prediction 3 – mortality is male, not female, biased

##### Proportion tests on cause of deaths and gender bias in stillbirths

To test the prediction that mortality is male-biased we first assessed sex-dependent trends in cause of death in a subset of the sample (*n* = 155) for which we had accurate reporting of cause of death. A two-way Pearson *χ*^2^ test was performed to assess whether the distribution of cause of death differed by sex; owing to sample size considerations this test was performed on the juvenile cohort as a whole. If infanticide is an important mechanism underlying skewed sex ratios, yet a population is unwilling to disclose such behaviour, more females may simply be reported as stillborn. Therefore, an exact binomial test was also performed to assess whether the proportion of stillbirths (*n* = 20) differed by sex.

##### Hazard analysis on juvenile mortality

Finally, a hazard analysis was performed to assess whether the proportional hazard of death across the juvenile period differed by sex. A multilevel Cox regression model was run (using the R package *survival*), in which all offspring of known sex (*n* = 623; miscarriages were excluded but stillborns included as sex was reported) were entered into the model and their survival duration in months assessed – for offspring who were alive at the time of data collection, data was right censored at either their current age or at 192 months (i.e. 16 years) to indicate the end of parental investment. Offspring (*n* = 623) were clustered by mother (*n* = 126) and camp (*n* = 12), resulting in a population-averaged model. Model selection based on Akaike's information criterion score was conducted to assess whether including year of the offspring's birth (to control for temporal effects (Ross et al. 2016), offspring's birth order (associated with mortality risk in other populations (Gellatly and Petrie 2017; Ross et al. 2016; Uggla and Mace 2016), or both caused less information to be lost – the model presented reflects the best fit, for other versions see the Supporting Information. Data processing, analysis and plotting were conducted in R version 3.1.2 (R Core Team 2012).

## Results

### Describing sex ratios in the Agta

**Figure 1.**
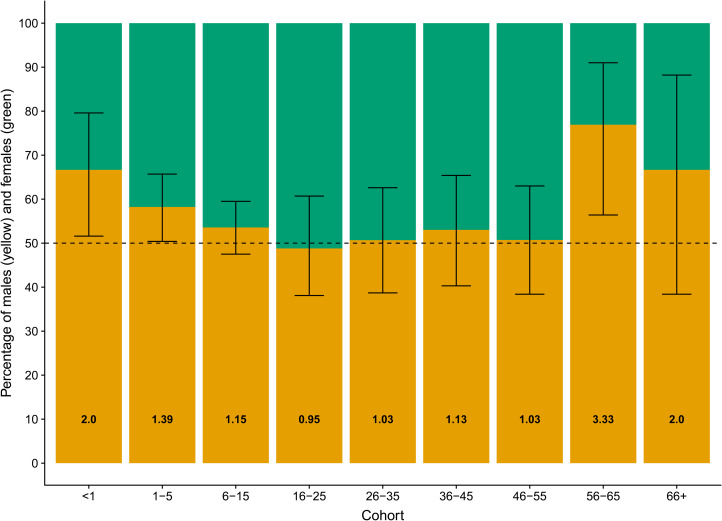
The percentage distribution of Agta males and females across age cohorts. Yellow (bottom) portion of the bars represents males; the green (top) portion represents female. The hashed line represents a balanced sex ratio and the numbers at the bottom of the bars are the sex ratios of each age group. Error bars are 95% confidence intervals of the observed proportions based on two-tailed binomial proportion tests. Sample sizes are as follows: <1, *n* = 48; 1–5, *n* = 170; 6–15, *n* = 280; 16–25, *n* = 168; 26–35, *n* = 73; 36–45, *n* = 66; 46–55, *n* = 69; 56–66, *n* = 26; and 66+, *n* = 15.

The sex ratios by age cohorts from 2013–2014 are presented in Figure 1; the sex ratio is significantly different from 1:1 in the <1 and 1–5 year cohorts, after which the sex ratio remains largely balanced in reproductively aged cohorts. The overall skew of the <16s was significantly male biased at 1.29 [*n* = 281:217, males = 0.564, 95% CI (0.520, 0.607)]. The sex ratio was *greater* than 1.07 (proportion = 0.517) in the <1s (*n* = 32:16, males = 0.67, LCI = 0.539 one-tailed). Furthermore, while a small sample, in the 16 births that occurred during fieldwork there was a male bias of 3.00, significantly exceeding 1.06 (*n* = 12:4, males = 0.75, LCI = 0.516 one-tailed), without any occurrence of female-biased infanticide. Full proportion test results can be seen in Table S2.

**Figure 2.**
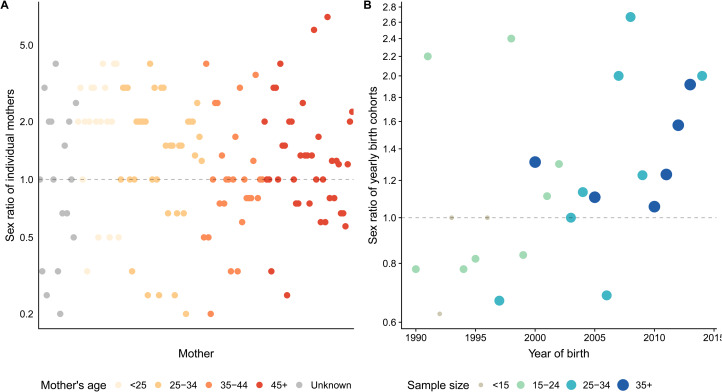
(a) The sex ratio of offspring born to individual mothers – here each dot is a mother, the age of whom is represented in shading. Only mothers with two or more offspring are included. (b) The sex ratio of birth cohorts from 1990 to 2014. Sample size is indicated by dot size – the larger the dot, the larger the sample. Accuracy is increased from the 2000s onwards as the size of the birth estimates is closer to the expected 35–40 births per year. The dashed line represents a balanced sex ratio. The *y-*axis is log transformed owing to the nature of the distribution of sex ratios.

Using reproductive histories, there was a trend for individual mothers to give birth to more boys than girls; of mothers who had given birth to at least 2 infants, 75 women had male-biased sex ratios, 22 balanced and 43 female-biased (Figure 2a). This relationship appears uninfluenced by maternal age, suggesting a lack of temporal trends, including under-reporting by older mothers. Older mothers, who on average had more children, also did not appear to have more balanced ratios (the sex ratio plotted against number of births, irrespective of age, can be seen in Figure S1).

There is also a trend for yearly birth cohorts to be male biased, at least since 1990 (1.22, Figure 2b): 15 male-biased, 3 balanced, 7 female-biased. Birth cohorts were more likely to be male-biased in more recent years, when sample sizes are also larger and closer to capturing the total number of births in a year. Figure 2b also highlights the issue of sample sizes diminishing over time when assessing yearly birth cohort sex ratios retrospectively. Nevertheless, based on both census and retrospective data there is clear reason to consider the Palanan Agta sex ratio to be male-biased in the early juvenile period.

### Prediction 1 – sex ratios become decreasingly male biased

#### Hypothesis tests using census data

Pearson *χ*^2^ tests find the proportion of males to be significantly higher in the <1 year cohort (0.667) compared with the 16–25 years cohort (0.488) [Pearson *χ*^2^ (d.f. = 1, *n* = 216) = 4.777, *p* = 0.029], supporting the hypothesus that a reduction in sex ratio occurs by adulthood; all other comparisons were non-significant (for full results see Table S3).

#### Illustrating differential mortality patterns on sex ratios trends

**Figure 3.**
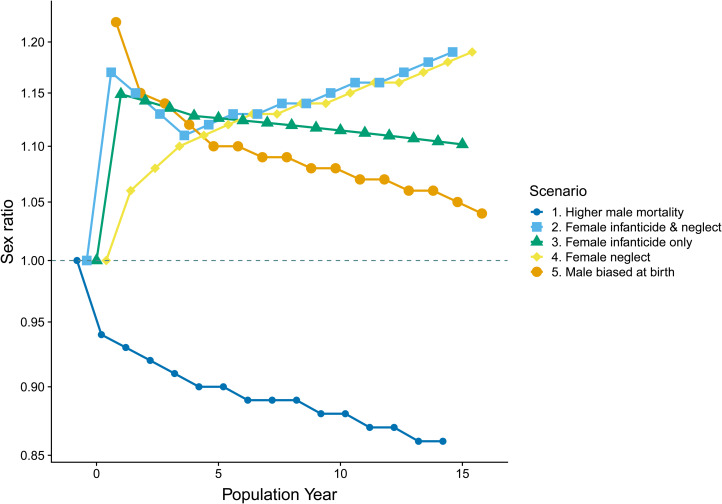
Population sex ratio by age cohort in a hypothetical population under differing sex-specific mortality scenarios. For full details see the Supporting Information. Lines have been dodged to assist viewing when there is significant overlapping. The dashed line represents a balanced (1:1) sex ratio. The *y-*axis is log transformed owing to the nature of the distribution of sex ratios.

The hypothetical sex ratios resulting from our five mortality scenarios are presented in Figure 3. When comparing the observed sex ratios across Agta juvenile age cohorts (see Figure 1 – we do not map them onto the model populations to avoid conflating period and cohort data) with the hypothetical sex ratios it is apparent that the scenarios based on systematic female neglect (scenario 4) and female infanticide with additional neglect (scenario 2) *do not* match the trajectory of the Agta sex ratios. The Agta ratios start high and then progressively decrease, best matching the line illustrating a male-biased sex ratio from birth (scenario 5). The female infanticide only (scenario 3) and higher male mortality ‘baseline’ (scenario 1) ratios also decline across age cohorts; however, the sex ratio of 3.00 for the subset of ‘witnessed’ births (*n* = 16) points against scenario 3 and the Agta sex ratios do not become female biased (scenario 1). Thus, the patterns derived from these scenarios are supportive of Agta sex ratios being male biased at birth.

### Prediction 2 – females do not receive less parental investment

Regression analysis indicates that there is no sex bias in the amount of care children receive from their parents when controlling for child age (Tables S4–6, Figure S2). While younger children interact significantly more with parents, the sex of the child has no effect, irrespective of the type of investment (high, low or total investment interactions) or source of investment (both parents combined, mother or father). Additionally, Wilcoxon rank sum tests found no significant differences between the levels of parental investment received by full brothers and sisters (Table S7).

### Prediction 3 – mortality is male, not female, biased

#### Proportion tests on cause of deaths and gender bias in stillbirths

While disease-related mortality was equally likely in both sexes, females were less likely to die of non-disease causes than males: Pearson *χ*^2^ (d.f. = 1, *n* = 155) = 4.726, *p* = 0.030 (Table 3). No difference was found between the proportions of males (*n* = 13) and females (*n* = 7) reported to have been stillborn (males = 0.65, LCI = 0.44 one-tailed).

**Table 3.** The distribution of the deaths of offspring reported by Agta mothers by sex

Cause of death	Age cohort									
	<1 year			1–5 years			6–15 years			Overall ratio
	Male *n*	Female *n*	Ratio	Male *n*	Female *n*	Ratio	Male *n*	Female *n*	Ratio	
Disease	24	32	0.75	22	15	1.47	16	14	1.14	1.02
Non-disease	6	2	3.00	12	3	4.00	5	4	1.25	2.56
**All causes**	**30**	**34**	**0.88**	**34**	**18**	**1**.**89**	**21**	**18**	**1**.**17**	**1**.**2**

#### Hazard analysis on juvenile mortality

In the best fit model, for every month after birth until the age of 16 years (192 months), the hazard of death was 56% higher for males compared with females [hazard ratio = 1.557, *p* = 0.011, 95% CI (1.113, 2.178)], after controlling for offspring's year of birth. Neither offspring's birth order or year of birth was a significant predictor. For full results see Table S8.

## Discussion

The juvenile sex ratio in the Palanan Agta is male biased; however, contrary to what has commonly been assumed, we find no evidence that females experience higher levels of juvenile mortality. The hazard of death across the period of parental investment was not higher for females, and females were not more likely to die from either disease- or non-disease-related causes of death. Our analysis of detailed Agta childcare data further finds no evidence that male offspring received preferential treatment. Further, running counter to the suggestion of female-biased mortality is the finding that Agta sex ratios did not increase across the juvenile period, as would be expected if female deaths outstripped those of males. Taken together, these results are not supportive of excessive female mortality. Rather, our results indicate that, in the Agta, males suffer higher levels of mortality during the juvenile period. Males were consistently just over 0.5 times as likely to die in any given month than females, until the age of 16 years. Furthermore, males were more likely to die from non-disease-related causes of death – with a sex-specific mortality ratio of 2.56.

In line with findings of higher male mortality, sex ratios derived from the Agta census data decline across the juvenile cohorts, reaching equilibrium by the end of parental investment. The observed sex ratios in the Agta best mirror the sex ratios predicted by a mechanism entailing either pure female infanticide followed by equal investment in female infants or a biological mechanism acting *in utero* to bias sex ratios in favour of males at birth – both of which predict that sex ratios will decline after infancy owing to male-biased juvenile mortality. Given that female infanticide reflects an active preference for males, it seems unlikely that it would exist in isolation from continued female neglect. Furthermore, given that we find no evidence of parental preference and no differences in the number of reported stillborns by sex and male-biased juvenile mortality, we consider a behavioural explanation for the observed patterns hard to reconcile. Combined with the tendency for individual mothers to report having more sons than daughters and there being more males in the witnessed births and <1 year group, we suggest that it is plausible that the Agta sex ratio at birth, in the recent past at least, is substantially male biased as a result of a biological mechanism adjusting the primary and/or secondary sex ratio.

Evidence supports a variety of proximate biological mechanisms by which sex ratios may be skewed by birth. For instance, the sex ratio at conception may be influenced paternally, as a result of an excess of sperm carrying X- or Y-bearing chromosomes, which is influenced by the ratio of testosterone to gonadotrophin (for a review see Navara 2013). The majority of the literature, however, focuses on the maternal role in sex manipulation. For instance, the viscosity of cervical mucus may be increased by greater production of estrogen, aiding X-bearing sperm (Navara 2013). More recent work has estimated the sex ratio at conception to be balanced in a subsample of US conceptions, after which differential mortality during gestation determines the sex ratio at birth (Orzack et al. 2015).

After conception, *in vitro* studies have shown that glucose has a different influence on the development of male and female blastocysts, with higher levels of glucose inhibiting the development of female conceptuses (for a review see Cameron 2004). Stress is associated with higher circulating levels of glucose, thus maternal stress could result in a greater production of male offspring (Cameron 2004), pointing to a means by which extrinsic risk may influence secondary sex ratios. Further, a recent study of gestational mortality and the trajectory of sex ratios across gestation in the US found total female mortality to be higher (Orzack et al. 2015). In support of facultative adjustment, from the expanding blastocyst phase there is sexual dimorphism in interferon-tau expression (Larson et al. 2001), the molecule which signals pregnancy to the mother (Bazer et al. 1997); this may provide plasticity in the selection of embryos (Flint et al. 1997; Larson et al. 2001).

In the case of the Agta, our results are suggestive of a Fisherian mechanism, with an early sex ratio bias towards males countering male-biased juvenile mortality. Male children seem more likely to die from events primarily beyond parental control, in line with the existing literature indicating male offspring to be subject to greater extrinsic mortality risk (Wells 2000). Thus, by producing more males at birth, parents are equalising their investment between the sexes as males are the least costly sex (Fisher 1930). While we did not test whether male offspring are more cooperative than females, and so cannot empirically rule this possibility out, the lack of bias in parental investment points against this explanation. The fact we find sex ratios balanced at the end of parental investment also runs counter to this position – Agta parents, by whatever mechanism, do not manipulate sex ratios such that they have more male offspring by the time they reach their peak productivity, as would be expected if males were the ‘most valuable’ sex.

Although the evidence presented here best supports a Fisherian explanation, rather than a behavioural mechanism, evolutionary-based explanations for sex ratios are not mutually exclusive (Sieff et al. 1990); individuals *within* a population experience different conditions and it should not be assumed all individuals will pursue the same strategy. It is a limitation of our study that we must rely on cross-sectional cohort data and retrospectively reported reproductive histories that cannot account for longitudinal maternal condition, and so are unable to assess the strategies of individual mothers (West et al. 2002). We expect any biological mechanism that adjusts at-birth sex ratios to be universal in humans, thus all individuals (or most likely, all females) are capable of facultatively producing more offspring of a given sex, where this would benefit their fitness. However, it does not follow that the majority of women in *all* foraging populations will pursue a strategy of having more males. Variations in juvenile mortality risk and/or the relative benefits of having males vs females owing to their productivity, for instance, are expected to lead to variations in the reproductive strategies of individuals within and between populations.

This latter point is illustrated by the differences and similarities between the Agta living in Palanan and Casiguran. In Casiguran Headland (1989) reports a biased juvenile sex ratio during two population censuses (1.22 in 1977 and 1.45 in 1984), and similar to Palanan, owing to male-biased juvenile mortality (Ross et al. 2016), sex ratios decline over time from infancy (1.14 and 1.68 in 0–4 year olds) to adulthood (0.9 and 0.83 in the >15s). However, in 1977 no cohort was statistically different from 1:1, while in 1984 the juvenile cohorts were skewed and the adult cohort balanced (Headland 1989). Furthermore, retrospective reproductive histories from Casiguran showed skewed sex ratios (0.515); however, the 95% CI overlapped with 0.5 (0.49, 0.53) (Headland et al. 2011). A variety of social factors potentially underlie these differences. The Agta of Casiguran have experienced relatively more social and economic disruption by loggers, miners and settlers resulting in their becoming peasant labours, with low status and low survival rates (Headland 1988). One consequence of this was the advent of hypergyny, with Agta women marrying ‘higher’ status non-Agta males to ensure access to farm land (Early and Headland 1998; Ross et al. 2016), a trend which has not been reported in Palanan. When hypergyny occurs, the relative fitness benefits of daughters may be heightened, leading parents to increase investment in female offspring (Bailey 1988; Bereczkei and Dunbar 1997; Cronk 1991; Hewlett 1993), indicated by greater investment (inferred from differential mortality) in first-born daughters compared with first-born sons in Casiguran (Ross et al. 2016). Consequently, the Agta in Casiguran may be facing opposing forces – increased male mortality making sons ‘cheaper’ vs hypergyny making daughters more advantageous – leading to a divergence from patterns observed in Palanan.

Given our small sample size, we do not wish to emphasise that the Palanan Agta are ‘special’ or overemphasise the definitiveness of our results. Future comparative studies with significantly more statistical power and robust design should further explore the relationship between sex ratios at birth and sex-specific mortality patterns. Nonetheless, while the Agta are a small population, the male bias in infancy was significantly higher than the global male bias [itself suggested to be compensating for higher male mortality (Fisher 1930; Mace and Jordan 2005)], which is unsurprising given the high extrinsic risks facing Agta boys. Therefore, this case study offers support to an adaptive hypothesis and acts as the first step in stimulating future research.

By taking a multifaceted approach, we hope to have provided sufficient cause for looking more critically at behavioural-based assumptions regarding male-biased sex ratios in foraging populations and, in the absence of evidence, avoid the further invocation of gendercide. *A priori* assumptions of female infanticide and/or neglect not only unnecessarily stigmatise groups, but also mean that interventions aimed at improving child outcomes are potentially misdirected (Shenk et al. 2014). Thus, we emphasise the cautionary point that, without exploring, in detail, mortality trends and parental investment strategies in a given population, it is impossible to comment on the behavioural or biological mechanisms behind sex ratio trends.

The Palanan Agta display male-biased sex ratios; however, we find no evidence of biased parental investment or higher female mortality – in fact mortality risk is male biased, as a result of an excess of non-disease related deaths. Sex ratios decline across age cohorts, becoming balanced by the end of parental investment, further indicating that male juveniles die at higher rates and suggesting that Agta mothers are responding, in accordance with the predictions of Fisher (1930), by adaptively adjusting their sex ratios at birth to equalise parental investment across the sexes. Thus, the Agta appear provide evidence to in support of adaptive manipulation of sex ratios, without recourse to active, or passive, gendercide.

## Data Availability

The authors declare that the data and code supporting the findings of this study are available and can be found at the OSF project site: https://osf.io/72umn/

## Supplementary material

For supplementary material accompanying this paper visit http://dx.doi.org/10.1017/ehs.2019.5. click here to view supplementary material
