# Supplementary material for: Why so many Agta boys? Explaining ‘extreme’ sex ratios in Philippine foragers
Source: Evol Hum Sci. 2019 May 30;1:e5. doi: 10.1017/ehs.2019.4 (PMC10427305; doi:10.1017/ehs.2019.4)
Supplement: Supplementary file 1 [file S2513843X19000045sup001.pdf]

Why so many Agta boys? Explaining 'extreme' sex ratios in Philippine foragers. Supplementary Information

Ethnographic detail

There are around 1,000 Palanan Agta living in Isabela Province, located in the northeast of Luzon, in the Philippines. The Agta reside in the Northern Sierra Madre Natural Park (NSMNP), a protected area that consists of a mountainous tropical rainforest and includes the coastal beaches, coral reefs and the marine eco-system of the Pacific Ocean.

On average 19.6% of food is produced from cultivation while the reminding 80.4% is produced by foraging activities (fishing, hunting and gathering). Further breaking this down into food groups (meat, vegetable and fruit and rice) we find that average meat consumption (primarily fish and other marine resources) equals 0.3 ± 0.1 of the total diet, compared to vegetables and fruit with consist of 0.2 ± 0.15 of the diet. While rice makes up 44% of the diet, there is significant variance in this figure as it is dependent on amount of time in wage labor and cultivation: as a proportion of the diet, some households consumed little rice (minimum = 12.5%), while the most extreme households had a diet consisting of 75% rice. Similarly, households involved in a high proportion of foraging (more than 75% of food production activities) consume more honey and less rice than individuals who spend more time in cultivation and wage labor. Likewise, individuals living in settled camps produce 78.9% of their food by foraging while individuals in mobile camps produce 90.9% of their food from hunting and gathering. The Agta rely heavily on foraging modes of subsistence (76.5%) versus non-foraging activities (23.5%). Time spent in cultivating their own land (clearing, planting, tending and harvesting) ranged between 4-11% for males and 3-13% for females in three different environments (coastal, interior and watershed). From this work, the crop yielded an average of 283 kg of rice per household, enough for 113 days, given an average daily rice consumption of 2.5kg.

Extended Methods

Focal follows protocol

A focal child is observed for 12 hours over several days to ensure a range of activities are captured; this 12-hour period is broken into three 4-hour intervals (6:00 – 10:00, 10:00 – 14:00 and 14:00 – 18:00) during which who the child is interacting with and what type of interaction this is recorded every 20 seconds (observe for 20 seconds, record for 10 seconds) within a three-meter radius. These 4-hour intervals were conducted on non-consecutive days to reduce any sampling bias (for example, the father being out of camp for those two days). A 15-minute break was taken every hour, thus in total each child was observed for approximately 9 hours, producing 1,080 observational points per child over three days. The following pieces of information are recorded for up to six individuals within three-meter proximity to the focal child: high investment activity (carry, feed, groom, medical attention, teach and play); and low investment activity (talking to, watching, and being just in proximity)

Modelled sex ratio distributions

It is important to emphasise these scenarios are intended to be purely illustrative. There is no such thing as a 'model' hunter-gatherer population, nor do we expect there to be a 'pure' population in which there is not some form of facultative mechanism for altering sex ratios in response to environmental circumstances already in activation; therefore, we have not used observed data. However, the mortality rates used to construct the sex ratios for our model population under differing mortality scenarios are within the bounds of possibility, given the level of mortality documented in foraging populations; for instance, based on figures presented in Kelly (2013) the average mortality under age 15 years is 35.3% (n = 27) and sex differences in mortality have been found to differ by as much as 14%. We also apply a pattern of mortality typical of 'non-industrialised' populations, in which a large proportion of deaths occur in infancy and the early juvenile period (Blurton Jones 2016); for example, from the Hadza single year life tables presented by Blurton Jones (2016) 51.8% of deaths occurring by year 15 had occurred by year 1 and 80.5% by year 4. The equivalent figures for the Agta are 48.8% and 84.5% (Page et al., 2016).

Each population starts with 1000 births and then experience one of five sex specific mortality scenarios:

1) Higher male mortality (this is the 'baseline' scenario reflecting naturally higher male mortality and no compensatory mechanisms):

- By year 15, 36% of the total population has died
- By year 15, 41% of the male population has died
- By year 15, 31% of the female population has died
- 50% of deaths occur by year 1
- 75% of deaths occur by year 4

2) Female infanticide + neglect (males are preferentially treated, some more males survive due to redirected investment, a lot more females die due to neglect, the majority of the excess female deaths occur in the first year)

- Males mortality improves by 5% from the base population
- 15% more females die than in the base population
- 50% of male deaths occur by year 1, 75% of male deaths occur by year 4
- 65% of female deaths occur by year 1, 75% of female deaths occur by year 4
- By year 15, 41% of the total population has died
- By year 15, 36% of the male population has died
- By year 15, 46% of the female population has died

3) Female infanticide only (females experience excess death as a result of selective infanticide in the first year, otherwise baseline conditions apply)

- By year 15, 43% of the total population has died
- By year 15, 41% of the male population has died
- By year 15, 46% of the female population has died
- 50% of male deaths occur by year 1
- 75% of male deaths occur by year 4
- 65% of female deaths occur by year 1

- 90% of female deaths occur by year 4

4) Female neglect (males are preferentially treated, some more males survive due to redirected investment, a lot more females die due to neglect)

- Males mortality improves by 5% from the base population
- 15% more females die than in the base population
- 50% of deaths occur by year 1
- 75% of deaths occur by year 4
- By year 15, 41% of the total population has died
- By year 15, 36% of the male population has died
- By year 15, 46% of the female population has died

5) Male biased at birth (males have a naturally higher death rate, more males are born)

- By year 15, 36% of the total population has died
- By year 15, 41% of the male population has died
- By year 15, 31% of the female population has died
- 50% of deaths occur by year 1
- 75% of deaths occur by year 4
- 20% more males are born than females

Descriptive statistics

Parental investment

Over the periods of time the children were observed, on average they were found to be in interaction with at least one parent 50% of the time; interactions involving activities requiring low levels of investment were more common than those requiring high investment, occurring on average 37% of the time children were observed compared to 25%. Finally, children were seen interacting with their mothers 69% of the time, compared with their fathers who they interacted with 31% of the time, on average.

Table S1 Child-parent interaction data (n = 35)

Variable	Min	Max	Mean	SD
**Age**	0.08	5.92	2.49	1.77
**Total mother observations**	0.09	0.99	0.69	0.24
**Total father observations**	0.02	0.67	0.31	0.16
**Maternal high investment**	0.00	0.60	0.24	0.20
**Maternal low investment**	0.09	0.85	0.46	0.18
**Paternal high investment**	0.00	0.10	0.03	0.03
**Paternal low investment**	0.02	0.66	0.28	0.16
**Total interactions**	0.13	0.78	0.50	0.16
**Total low investment interactions**	0.07	0.71	0.37	0.15
**Total high investment interactions**	0.07	0.55	0.25	0.12

Extended Results

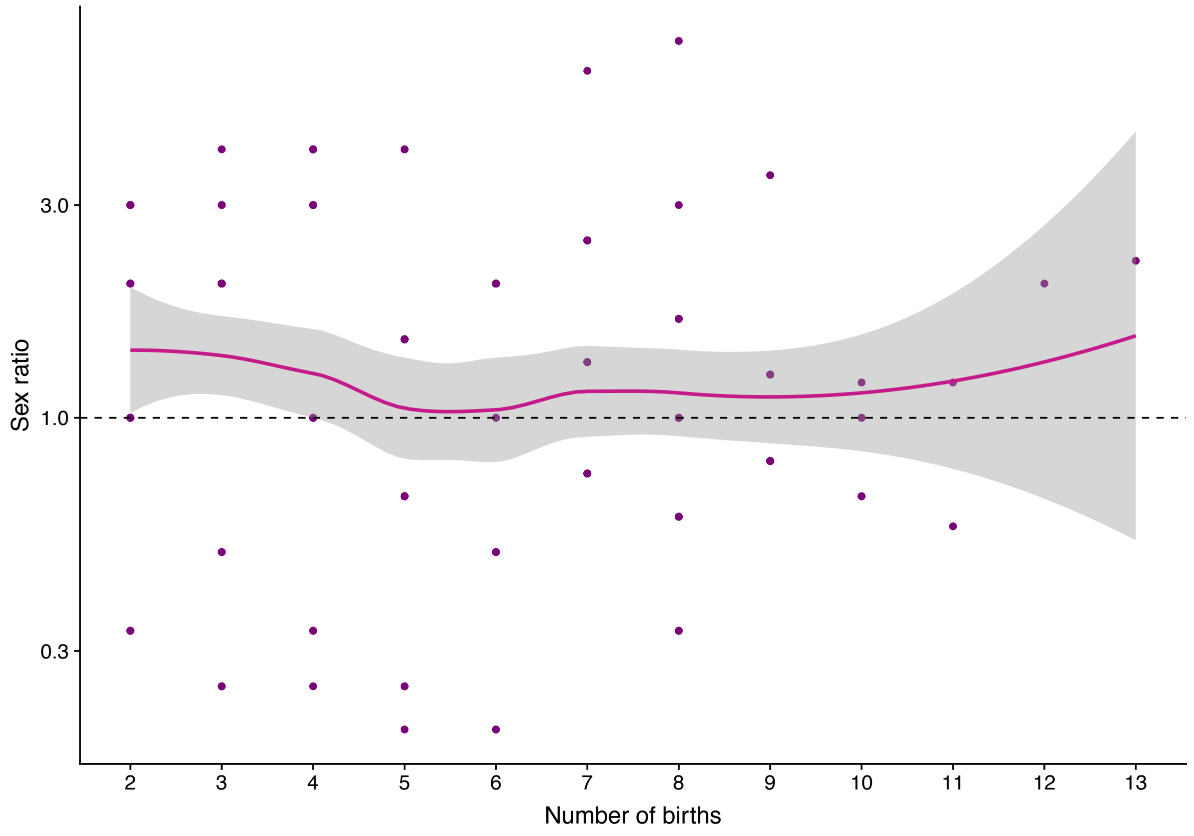

Figure S1: The sex ratio of offspring (alive or dead) born to a mother by her number of births so far. If the chance of having an infant of a given sex is 50:50, then the more births a mother has, the closer her sex ratio should be to 1.00. Here each dot represents multiple women at each number of births (range 1-19). The 95% CI is in grey and widens at 10-13 births these consist of much smaller sample sizes of mothers (10 – n = 5; 11 – n = 2; 12 and 13 – n = 1). The dashed line represents a balanced sex ratio. The y-axis has been log transformed.

Table S2 Full binomial exact test results for each cohort. Reference proportion to which the *p* value refers is 0.5 (i.e. a sex ratio of 1:1). Confidence intervals (CI) which do not overlap with 0.5 are in bold, results which do not overlap with 0.517 (i.e. a sex ratio of 1.07) are in italics – also of interest is whether CIs overlap 0.515 (i.e. 1.06) and 0.512 (i.e. 1.05).

Cohort	Direction	#m/#f	n	sex ratio	Prop male	95% CI	*p*
Observed*	One-tailed	12/4	16	3	0.75	**0.516, 1.000**	**0.038**
Observed*	Two-tailed	12/4	16	3	0.75	0.476, 0.927	0.077
Under 1	*One-tailed*	*32/16*	*48*	*2*	*0.667*	***0.539, 1.000***	***0.015***
Under 1	*Two-tailed*	*32/16*	*48*	*2*	*0.667*	**0.516, 0.796**	**0.029**
1-5	*One-tailed*	*99/71*	*170*	*1.39*	*0.582*	***0.517, 1.000***	***0.019***
1-5	Two-tailed	99/71	170	1.39	0.582	**0.504, 0.657**	**0.038**
6-15	One-tailed	150/130	280	1.15	0.536	0.485, 1.000	0.128
6-15	Two-tailed	150/130	280	1.15	0.536	0.475, 0.595	0.232
16-25	Two-tailed	40/41	81	0.95	0.494	0.381, 0.607	1
26- 35	Two-tailed	37/36	73	1.03	0.507	0.387, 0.626	1
36-45	Two-tailed	35/31	66	1.13	0.53	0.403, 0.654	0.7122
46-55	Two-tailed	35/34	69	1.03	0.507	0.384, 0.630	1
56-65	Two-tailed	20/6	26	3.33	0.769	***0.564, 0.910***	***0.009***
66 +	Two-tailed	10/5	15	2	0.667	0.384, 0.882	0.302
Under 16	One-tailed	281/217	498	1.29	0.564	***0.526, 1.000***	***0.002***
Under 16	Two-tailed	281/217	498	1.29	0.564	***0.520, 0.607***	***0.004***
Full sample	Two-tailed	500/415	915	1.2	0.547	**0.514, 0.579**	**0.006**

Table S3 Chi-square and significance values from Pearson Chi-square tests assessing differences in the distribution of the sexes by age cohort. The bottom of the matrix (grey

boxes) reports the chi-square statistic, the top (white boxes) the *p*-value; α <0.05 indicated in bold.

	<1 (n=48)	1-5 (n=170)	6-15 (n=280)	16-25 (n=168)
**<1 (n=48)**	-	0.292	0.092	**0.029**
**1-5 (n=170)**	1.110	-	0.335	0.082
**6-15 (n=280)**	2.845	0.931	-	0.329
**16-25 (n=168)**	**4.777**	3.018	0.954	-

Table S4 Multilevel results for relationship between total parental interactions (both maternal and paternal) with their children dependent on the sex of that child, controlling for age (*n* = 32).

	Total interactions	High investment	Low investment

**Parameter**	***B***	***p***	**CI**	**CI**	***B***	***p***	**CI**	**CI**	***B***	***p***	**CI**	**CI**
Intercept	0.558	<0.001	0.465	0.650	0.353	<0.001	0.284	0.418	0.312	<0.001	0.219	0.404
Female child	0.060	0.170	-0.032	0.149	0.049	0.101	-0.012	0.060	0.031	0.503	-0.064	0.125
Child age	-0.03	0.008	-0.052	-0.005	-0.044	<0.001	-0.046	-0.024	0.021	0.086	0.044	0.046
**Random effects**												
Household variance	0.02 (65.4%)				0.01 (61.5%)				0.0096 (46.4%)			
Dyadic variance	0.01 (34.6%)				0.004 (38.5%)				0.0111 (53.6%)			
**R^2_m**	0.137				0.401				0.070			
**R^2_S**	0.702				0.770				0.502			

Table S5 Multilevel results for relationship between maternal interactions with their children dependent on the sex of that child, controlling for age (*n* = 33).

	**Total interactions**				**High investment**				**Low investment**			
**Parameter**	***B***	***p***	**CI**	**CI**	***B***	***p***	**CI**	**CI**	***B***	***p***	**CI**	**CI**
Intercept	0.841	<0.001	0.717	0.966	0.458	<0.001	0.382	0.532	0.398	<0.001	0.283	0.519
Female child	0.048	0.420	-0.084	0.170	0.045	0.219	-0.030	0.067	-0.020	0.744	-0.145	0.102
Child age	-0.065	<0.001	-0.097	-0.033	-0.095	<0.001	-0.115	-0.074	0.027	0.104	-0.008	0.060
**Random effects**												
Household variance	0.03 (60.2%)				0.007 (48.2%)				0.004 (13.7%)			
Dyadic variance	0.02 (39.8%)				0.007 (51.8%)				0.027 (86.3%)			
**R^2_m**	0.259				0.693				0.077			
**R^2_S**	0.705				0.841				0.203			

Table S6 Multilevel results for relationship between paternal interactions with their children dependent on the sex of that child, controlling for age (*n* = 33).

	Total interactions				High investment				Low investment			
**Parameter**	*B*	*p*	CI	CI	*B*	*p*	CI	CI	*B*	*p*	CI	CI
Intercept	0.273	<0.001	0.164	0.371	0.025	0.009	0.007	0.043	0.254	<0.001	0.146	0.352
Female child	0.069	0.131	-0.063	0.079	0.019	0.052	0.000	0.038	0.055	0.211	-0.050	0.079
Child age	0.008	0.505	-0.006	0.030	-0.002	0.445	-0.007	0.003	0.007	0.548	-0.006	0.030
**Random effects**												
Household variance	0.02 (61.4%)				NA				0.017 (67.3%)			
Dyadic variance	0.009 (38.6%)				NA				0.008 (32.7%)			
**R^2_m**	0.050				NA				0.032			
**R^2_S**	0.633				0.145				0.683			

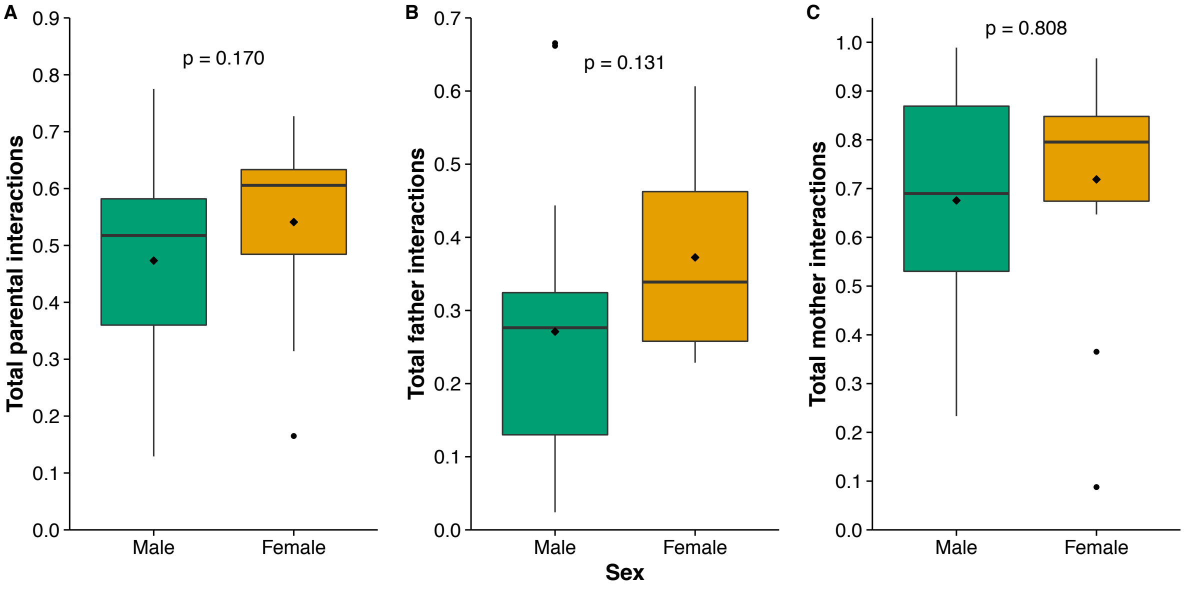

Figure S2 Boxplots demonstrating the relationship between parental investment and child sex for **A)** total parental interactions (*n* = 33 dyads, *n* = 22 mother-father pairs), **B)** total maternal interactions (*n* = 33 dyads, *n* = 23 mothers), and **C)** total paternal interactions (*n* = 32 dyads, *n* = 22 fathers). Diamond-shaped points represent the group mean, the lines the median. P-values are from the multilevel regression analysis reported in text.

Table S7 Results of Wilcoxon rank sum tests assessing the difference in parental investment received by brothers and sisters (sibling sets n = 5, children n = 12).

Investment source	Wilcoxon rank sum tests: W, *p*		
	**High investment interactions**	**Low investment interactions**	**Total investment interactions**
Parental investment	W = 14, p = 0.589	W = 15, p = 0.699	W = 11, p = 0.310
Maternal investment	W = 17, p = 0.937	W = 13, p = 0.485	W = 14, p = 0.589
Paternal investment	W = 21, p = 0.699	W = 12, p = 0.394	W = 14, p = 0.589

Table S8 Results of multilevel cox regression assessing the hazard of death overtime between birth and age 16 years of 623 offspring born to 126 mothers.

**Variable**		**Coef.**	**Robust S.E.**	***p***	**H.R.**	**95% C.I. of the H.R.**		**AIC**
						**Lower**	**Upper**	
Univariable model								
Sex	Male	0.412	0.166	**0.013**	1.510	1.09	2.094	1529.008
	Female (ref.)	-	-	-	-	-	-	
Multivariable model – Sex & year of offspring's birth								
Sex	Male	0.443	0.171	**0.01**	1.557	1.113	2.178	1491.721
	Female (ref.)	-	-	-	-	-	-	
Year of offspring's birth		0.004	0.010	0.664	1.004	0.985	1.024	
Multivariable model – Sex & birth order								
Sex	Male	0.414	0.187	**0.013**	1.513	1.087	2.103	1530.681
	Female (ref.)	-	-	-	-	-	-	
Birth order		0.020	0.047	0.667	1.021	0.930	1.120	
Multivariable model – Sex, year of offspring's birth, & birth order								
Sex	Male	0.444	0.171	**0.009**	1.559	1.116	2.178	1493.557
	Female (ref.)	-	-	-	-	-	-	
Year of offspring's birth		0.003	0.010	0.738	1.003	0.984	1.023	
Birth order		0.015	0.037	0.758	1.015	0.922	1.118	
